# Anti-Pathogenic and Immune-Modulatory Effects of Peroral Treatment with Cardamom Essential Oil in Acute Murine Campylobacteriosis

**DOI:** 10.3390/microorganisms9010169

**Published:** 2021-01-14

**Authors:** Markus M. Heimesaat, Soraya Mousavi, Dennis Weschka, Stefan Bereswill

**Affiliations:** Gastrointestinal Microbiology Research Group, Institute of Microbiology, Infectious Diseases and Immunology, Charité-University Medicine Berlin, Corporate Member of Freie Universität Berlin, Humboldt-Universität zu Berlin, and Berlin Institute of Health, 12203 Berlin, Germany; soraya.mousavi@charite.de (S.M.); dennis.weschka@charite.de (D.W.); stefan.bereswill@charite.de (S.B.)

**Keywords:** cardamom essential oil, enteropathogenic infection, *Campylobacter jejuni*, immune-modulatory effects, antipathogenic effects, microbiota-depleted IL-10^−/−^ mice, campylobacteriosis model, host–pathogen interaction, preclinical intervention study

## Abstract

Human infections with enteropathogenic *Campylobacter jejuni* (*C. jejuni*) including multi-drug resistant isolates are emerging worldwide. Antibiotics-independent approaches in the combat of campylobacteriosis are therefore highly desirable. Since the health-beneficial including anti-inflammatory and anti-infectious properties of cardamom have been acknowledged for long, we here addressed potential anti-pathogenic and immune-modulatory effects of this natural compound during acute campylobacteriosis. For this purpose, microbiota-depleted IL-10^−/−^ mice were orally infected with *C. jejuni* strain 81–176 and subjected to cardamom essential oil (EO) via the drinking water starting on day 2 post-infection. Cardamom EO treatment resulted in lower intestinal pathogen loads and improved clinical outcome of mice as early as day 3 post-infection. Furthermore, when compared to mock controls, cardamom EO treated mice displayed less distinct macroscopic and microscopic inflammatory sequelae on day 6 post-infection that were paralleled by lower colonic numbers of macrophages, monocytes, and T cells and diminished pro-inflammatory mediator secretion not only in the intestinal tract, but also in extra-intestinal and, remarkably, systemic organs. In conclusion, our preclinical intervention study provides the first evidence that cardamom EO comprises a promising compound for the combat of acute campylobacteriosis and presumably prevention of post-infectious morbidities.

## 1. Introduction

Human infections with pathogens from the *Campylobacter* clade including *Campylobacter jejuni (C. jejuni)* are emerging globally and are responsible for increasing health care and socioeconomic burdens [1,2]. In Europe, progressively increasing incidences of *Campylobacter* infections have been reported since 2008 and outnumber salmonellosis cases by approximately three-fold [3]. The route of *Campylobacter* transmission to humans is mostly food- or water-borne and occurs upon ingestion of contaminated raw or under-cooked meat products from livestock, particularly from poultry, or of bacteria-containing surface waters [4,5]. Following an incubation period of up to five days, infected individuals might complain about discomfort of varying degree depending on the arsenal of virulence factors expressed by the pathogen and the individual immune status. Patients usually present with typical symptoms of campylobacteriosis such as nausea, vomiting, headache, fever, abdominal cramps, and watery or even bloody diarrhea with mucous discharge [2,6]. In immune-competent subjects, only symptomatic treatment including substitution of fluids and electrolytes is indicated, and the disease usually resolves within two weeks without residues, whereas in immune-compromized patients, antibiotic treatment with macrolides or fluoroquinolones may be indicated. Post-infectious autoimmune diseases may occur weeks or months post-infection (p.i.) in rare instances, however, affecting the central nervous system (e.g., Guillain Barré syndrome), the joints (e.g., reactive arthritis) and the gastrointestinal tract (e.g., irritable bowel syndrome, coeliac disease, and chronic inflammatory bowel diseases) [2,7]. During the acute phase of the disease, innate and adaptive immune cell populations infiltrating the intestinal mucosa and lamina propria, epithelial cell apoptosis, ulcerations, erosions, and even tissue destruction can be observed, resulting in epithelial barrier dysfunction [8,9].

For a long time, analyses of *C. jejuni*–host interactions have been limited by a scarcity of reliable in vivo models. Conventional laboratory mice, for instance, are protected from pathogenic infection due to their complex gut microbiota composition providing an effective physiological colonization resistance [10,11]. Upon depletion of the microbiota, however, wildtype mice can be stably infected by the pathogen and display inflammatory immune responses, which are characterized by intestinal accumulation of macrophages, monocytes, T- and B-lymphocytes, and enhanced pro-inflammatory mediator secretion, leading to pronounced epithelial cell apoptosis [12]. Furthermore, toll-like receptor-4 (TLR-4) has been shown to be critically involved in mediating *C. jejuni* induced immunopathological responses [12]. In microbiota-depleted wildtype mice, however, typical overt macroscopic and clinical signs of human campylobacteriosis such as bloody diarrhea were lacking upon *C. jejuni* infection [12]. In this context, one needs to take into consideration that mice are much more resistant to bacterial lipopolysaccharide (LPS) and lipooligosaccharide (LOS) abundant in the cell walls of Gram-negative bacteria including *Campylobacter* as compared to humans [13]. This physiological LPS/LOS resistance of mice can be overcome, however, by deletion of the *il10* gene, for instance [14]. In fact, within six days following peroral *C. jejuni* infection, microbiota-depleted IL-10^−/−^ mice did not only display high pathogen loads in their intestinal tract, but also suffered from wasting and bloody diarrhea indicative for acute enterocolitis and succumbed to infection [15]. This acute *C. jejuni* infection and inflammation model has been proven suitable to test distinct pathogenic virulence factors such as proteases [16,17,18], flagellins, and adhesins [19], which also holds true for the evaluation of defined molecules and compounds, regarding their anti-pathogenic and immune-modulatory properties in preclinical intervention studies [20,21,22,23,24,25].

Cardamom (*Elettaria cardamomum* Maton belonging to the family of *Zingiberaceae*) constitutes a perennial herb that is traditionally used as aromatic spice, particularly on the Indian subcontinent, but also in distinct parts of Africa and Central America, and is called “The Queen of Spices”, since it is considered the third most precious spice after saffron and vanilla [26]. Both cardamom extracts and essential oils (EOs) have been applied as “natural products” for the treatment of several disorders including colds, bronchitis, asthma, flatulence, indigestion, and jaundice, and furthermore as stimulating agents in anorectic patients [27,28]. Studies addressing the health-beneficial potential of cardamom revealed anti-oxidant, anti-inflammatory, anti-microbial, and even anti-cancerogenic properties [29,30,31,32,33], which might be explained by the fact that the plant is a rich source of biologically active phenolic compounds, terpenoids, alkaloids, anthocyanins, and flavonoids [28,34,35].

Given the plethora of cardamom-associated health-beneficial effects during inflammatory and infectious morbidities, we investigated potential anti-pathogenic and immune-modulatory effects of peroral cardamom EO treatment during acute murine *C. jejuni-*induced enterocolitis in our present preclinical intervention study.

## 2. Materials and Methods

### 2.1. Ethical Statement

Mouse studies were approved by the local ethical committee (“Landesamt für Gesundheit und Soziales”, LaGeSo, Berlin; registration number G0104/19, approved on 15 July 2019) and performed according to the ARRIVE and European Guidelines for animal welfare (2010/63/EU). Animal welfare was monitored every day.

### 2.2. Microbiota Depletion in IL-10^−/−^ Mice

IL-10^−/−^ mice (in C57BL/6j background) were bred in the Forschungsinstitute für Experimentelle Medizin, Charité University Medicine Berlin, Berlin, Germany and kept in cages equipped with filter tops within an experimental semi-barrier under standard conditions. By the age of seven weeks, littermate mice of both genders were subjected to broad-spectrum antibiotics for commensal gut microbiota depletion for eight weeks, as described previously [12,36]. In brief, following transfer to sterile cages (maximum of four animals per cage) mice were treated with a mix of ampicillin plus sulbactam (1 g/L; Dr. Friedrich Eberth Arzneimittel, Ursensollen, Germany), imipenem (250 mg/L; Fresenius Kabi, Bad Homburg, Germany), ciprofloxacin (200 mg/L; Fresenius Kabi, Bad Homburg, Germany), metronidazole (1 g/L; B. Braun, Melsungen, Germany), and vancomycin (500 mg/L; Hikma Pharmaceuticals, London, UK), which were dissolved in the autoclaved tap water (ad libitum). In order to avoid contaminations, microbiota-depleted mice were maintained and handled under strict aseptic conditions and received autoclaved food and drinking water. To assure antibiotic washout, the antibiotic cocktail was replaced by autoclaved tap water three days before the *C. jejuni* infections. Virtual absence of commensal gut bacteria were surveyed by culture and culture-independent (i.e., 16S rRNA based) methods, as described previously [37].

### 2.3. Campylobacter jejuni Infection

After thawing from a stock, *C. jejuni* strain 81–176 was grown on columbia agar (with 5% sheep blood) and selective karmali agar plates (both from Oxoid, Wesel, Germany) in a microaerophilic atmosphere for 48 h (37 °C). Colonies were harvested from a densely grown bacterial cell layer with a sterile cotton swab and transferred to sterile phosphate buffered saline (PBS; Thermo Fisher Scientific, Waltham, MA, USA). Microbiota-depleted IL-10^−/−^ mice (four-month-old sex- and age-matched littermates) were infected perorally with 10^9^ colony forming units (CFU) of the pathogen on days 0 and 1 by gavage.

### 2.4. Cardamom Essential Oil Treatment

Cardamom EO was purchased from Sigma-Aldrich (Munich, Germany), dissolved in 0.05% carboxymethylcellulose (Sigma-Aldrich, Munich, Germany; in sterile phosphate buffered saline, PBS, Thermo Fisher Scientific, Waltham, MA, USA), and then added to autoclaved tap water to a final concentration of 924 mg/L. Mice were subjected to the peroral cardamom EO treatment from day 2 until day 6 p.i., via the drinking water (ad libitum). Given a daily drinking volume of approximately 5 mL and a mean body weight of 20 g, the daily cardamom EO dose infected mice received was 258 mg per kg body weight. Mock treated mice received vehicle dissolved in drinking water only (ad libitum).

### 2.5. Gastrointestinal C. jejuni Loads and Bacterial Translocation

*C. jejuni* loads were determined in fecal samples over time and on day 6 p.i. in gastrointestinal luminal samples taken from the colon, ileum, duodenum, and stomach, and furthermore, in ex vivo biopsies derived from the kidneys, lungs, liver, spleen, and mesenteric lymph nodes (MLN) by culture, as stated earlier [12,38]. Briefly, samples were homogenized in sterile PBS (Thermo Fisher Scientific, Waltham, MA, USA) with a pistill, serial dilutions streaked on columbia agar (with 5% sheep blood) and karmali agar (both from Oxoid, Wesel, Germany) and incubated in a microaerophilic atmosphere (48 h, 37 °C). The detection limit of viable *C. jejuni* cells was 100 CFU per g. For surveying *C. jejuni* bacteremia, approximately 200 μL cardiac blood were transferred to thioglycollate enrichment broths (BD Bioscience, Heidelberg, Germany) and streaked onto respective solid media after a seven-day incubation period (37 °C) [12,38].

### 2.6. Clinical Outcome

The clinical outcome of mice was quantitatively surveyed on a daily basis starting immediately before *C. jejuni* infection on day 0 by applying a standardized clinical score (maximum 12 points), addressing the clinical aspect/wasting (0: normal; 1: ruffled fur; 2: less locomotion; 3: isolation; 4: severely compromised locomotion, pre-final aspect), the abundance of blood in feces (0: no blood; 2: microscopic detection of blood by the Guajac method using Haemoccult, Beckman Coulter/PCD, Krefeld, Germany; 4: macroscopic blood visible), and stool consistency (0: formed feces; 2: pasty feces; 4: liquid feces), as described earlier [16].

### 2.7. Sampling Procedures

Mice were sacrificed by CO_2_ asphyxiation on day 6 p.i. Cardiac blood, ex vivo biopsies from kidneys, lungs, liver, spleen, MLN, ileum, and colon, as well as luminal samples from stomach, duodenum, ileum, and colon were derived under aseptic conditions. From each mouse, colonic samples were collected in parallel for subsequent microbiological and immunohistopathological analyses. The small intestinal and colonic lengths were measured with a ruler.

### 2.8. Histopathology

Colonic ex vivo biopsies were immediately subjected to formalin fixation (5%) and embedded in paraffin. Following hematoxylin and eosin staining of 5 μm thin sections, the histopathological changes within the large intestines were assessed by light microscopy (100 × magnification) and quantified by using a standardized histopathological score as described earlier [39]: Score 1: minimal inflammatory cell infiltrates in the mucosa with intact epithelium. Score 2: mild inflammatory cell infiltrates in the mucosa and submucosa with mild hyperplasia and mild goblet cell loss. Score 3: moderate inflammatory cell infiltrates in the mucosa with moderate goblet cell loss. Score 4: marked inflammatory cell infiltration into in the mucosa and submucosa with marked goblet cell loss, multiple crypt abscesses, and crypt loss.

### 2.9. In Situ Immunohistochemistry

Apoptotic epithelial cells, macrophages and monocytes, T lymphocytes, regulatory T cells, and B lymphocytes were quantified following in situ immunohistochemical staining of 5 μm thin colonic paraffin with primary antibodies directed against cleaved caspase-3 (Asp175, Cell Signaling, Beverly, MA, USA, 1:200), F4/80 (no. 14-4801, clone BM8, eBioscience, San Diego, CA, USA, 1:50), CD3 (no. N1580, Dako, Glostrup, Denmark, 1:10), and FOXP3 (clone FJK-165, no. 14-5773, eBioscience, San Diego, CA, USA, 1:100), respectively, as reported earlier [40,41]. The average number of respective positively stained cells in each sample was assessed within six high power fields (HPF, 0.287 mm^2^, 400× magnification, light microscopy) by an independent and blinded investigator.

### 2.10. Pro-Inflammatory Mediators

Ex vivo biopsies were obtained from the terminal ileum and colon (longitudinally cut strips of approximately 1 cm^2^) and washed in PBS (Thermo Fisher Scientific, Waltham, MA, USA), and extra-intestinal ex vivo biopsies were derived from the kidney (one half after longitudinal cut), the lung, and the liver (approximately 1 cm^3^) and then transferred to 24-flat-bottom well culture plates (Thermo Fisher Scientific, Waltham, MA, USA) containing 500 μL serum-free RPMI 1640 medium (Thermo Fisher Scientific, Waltham, MA, USA) supplemented with penicillin (100 µg/mL) and streptomycin (100 µg/mL; Biochrom, Berlin, Germany), as stated earlier [12,36]. Respective culture supernatants and serum samples were tested for interferon-γ (IFN-γ), tumor necrosis factor-α (TNF-α), interleukin-6 (IL-6), and monocyte chemoattractant protein 1 (MCP-1) by the Mouse Inflammation Cytometric Bead Assay (CBA; BD Biosciences, Heidelberg, Germany) in a BD FACSCanto II flow cytometer (BD Biosciences, Heidelberg, Germany) after incubation at 37 °C for 18 h. Nitric oxide concentrations were determined by the Griess reaction [36,42], and systemic pro-inflammatory mediator secretion was assessed in serum samples.

### 2.11. Statistical Analyses

Medians and significance levels were calculated by using Prism, version 8 (GraphPad Software, San Diego, CA, USA). The Mann–Whitney test was applied for pairwise comparisons of not normally distributed data. For multiple comparisons, the one-sided ANOVA test with Tukey post-correction was used for normally distributed data, and the Kruskal–Wallis test with Dunn’s post-correction for not normally distributed data. Two-sided probability (*p*) values ≤ 0.05 were considered significant. Shown data sets were derived from four independent experiments with the following cohort sizes per individual experiment: naive (i.e., uninfected, untreated) microbiota-depleted IL-10^−/−^ mice: 4, 4, 4, 4. Cardomon EO treated and *C. jejuni* infected microbiota-depleted IL-10^−/−^ mice: 4, 4, 4, 4. Mock (i.e., vehicle) treated and *C. jejuni* infected microbiota-depleted IL-10^−/−^ mice: 4, 4, 4, 3.

## 3. Results

### 3.1. Gastrointestinal Pathogen Loads Following Oral Cardamom EO Application to C. jejuni-Infected Microbiota-Depleted IL-10^−/−^ Mice

**Figure 1:**
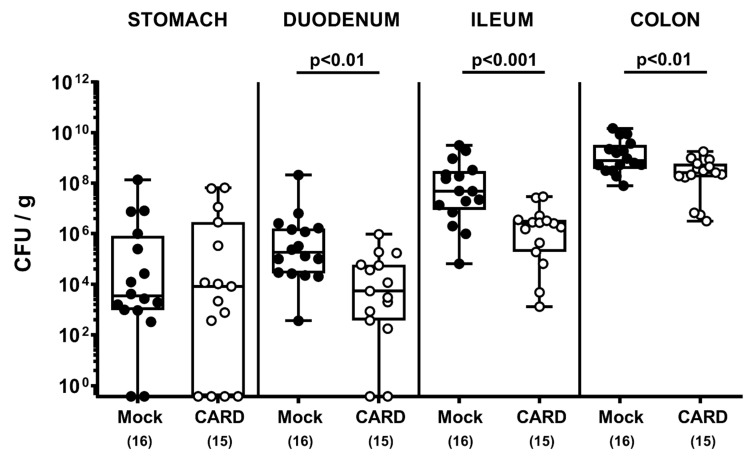
Gastrointestinal pathogen loads following oral cardamom essential oil application to *Campylobacter jejuni*- (*C. jejuni*-) infected microbiota-depleted IL-10^−/−^ mice. Microbiota-depleted IL-10^−/−^ mice were infected with *C. jejuni* strain 81–176 on days 0 and 1 by gavage and perorally challenged with cardamom essential oil (CARD; white circles) via the drinking water starting on day 2 post-infection, whereas mock control mice received vehicle (black circles). Gastrointestinal pathogen loads were determined by culture (in colony forming units (CFU) per g) on day 6 post-infection. The box plots indicating the 75th and 25th percentiles of the median (black bar within box), the total range, the levels of significance (*p* values) as calculated with the Mann–Whitney U test, and the number of analyzed mice (in parentheses) are shown. Data were pooled from four independent experiments.

Following initiation of oral cardamom EO treatment of *C. jejuni-*infected mice on day 2 p.i., we performed a kinetic survey of the fecal pathogen loads. On days 3, 4, and 6 p.i., cardamom EO treated mice harbored lower *C. jejuni* numbers in their intestines as compared to mock controls (*p* < 0.05–0.001; Supplemental Figure S1). Upon sacrifice, we additionally focused on the pathogen burdens alongside the gastrointestinal tract and observed approximately two orders of magnitude lower median *C. jejuni* loads in the small intestinal (i.e., in duodenum and ileum) and of one order of magnitude in the large intestinal (i.e., colon) lumen of cardamom EO as compared to mock treated mice on day 6 p.i. (*p* < 0.01–0.001), which was, however, not the case in the stomach (not significant (n.s.); Figure 1). Thus, cardamom EO treatment lowered the intestinal pathogen burdens in *C. jejuni*-infected microbiota-depleted IL-10^−/−^ mice.

### 3.2. Clinical Outcome Following Oral Cardamom EO Application to C. jejuni-Infected Microbiota-Depleted IL-10^−/−^ Mice

**Figure 2:**
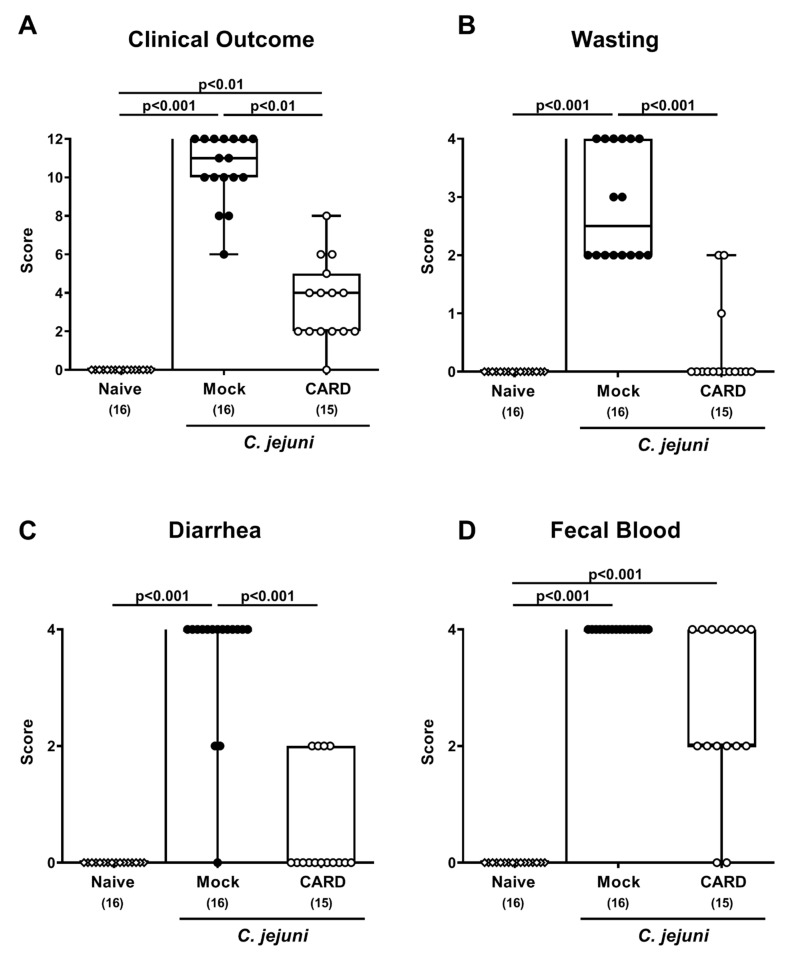
Clinical outcome following oral cardamom essential oil application to *C. jejuni*-infected microbiota-depleted IL-10^−/−^ mice. Microbiota-depleted IL-10^−/−^ mice were infected with *C. jejuni* strain 81–176 on days 0 and 1 by gavage and perorally challenged with cardamom essential oil (CARD; white circles) via the drinking water starting on day 2 post-infection, whereas mock control mice received vehicle (black circles). On day 6 post-infection the (**A**) clinical outcome as determined by (**B**) wasting, (**C**) diarrhea, and (**D**) fecal blood were quantitatively assessed by a clinical scoring system. Naive mice (open diamonds) served as untreated and uninfected controls. The box plots indicating the 75th and the 25th percentiles of the median (black bar within box), the total range, the levels of significance (*p* values), as calculated with the Kruskal–Wallis test, and Dunn’s post-correction and the number of analyzed mice (in parentheses) are shown. Data were pooled from four independent experiments.

When assessing the clinical outcomes in respective cohorts, cardamom EO treated mice displayed lower clinical scores as compared to mock control animals from day 3 until day 6 p.i. (*p* < 0.01–0.001; Supplemental Figure S2; Figure 2). Upon sacrifice, when mice from the mock cohort were suffering from severe pathogen-induced disease as characterized by wasting and bloody diarrhea, cardamom EO treated mice were far less clinically compromised and displayed, if at all, rather mild to moderate clinical signs on day 6 p.i. (Figure 2). Thus, cardamom EO treatment improved the clinical outcome in *C. jejuni-*infected IL-10^−/−^ mice.

### 3.3. Intestinal Lengths Following Oral Cardamom EO Application to C. jejuni-Infected Microbiota-Depleted IL-10^−/−^ Mice

**Figure 3:**
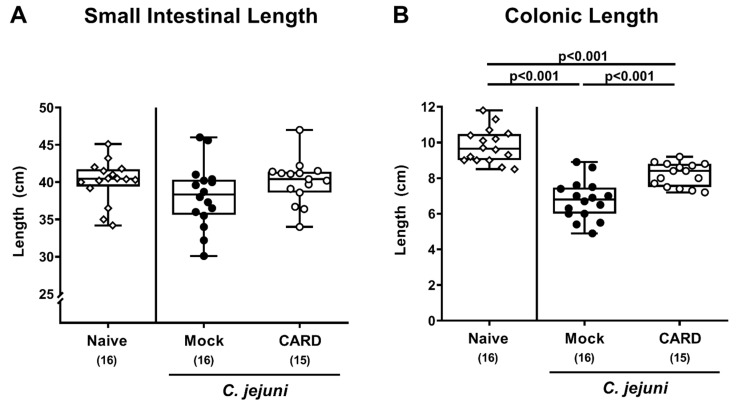
Intestinal lengths following oral cardamom essential oil application to *C. jejuni*-infected microbiota-depleted IL-10^−/−^ mice. Microbiota-depleted IL-10^−/−^ mice were infected with *C. jejuni* strain 81–176 on days 0 and 1 by gavage and perorally challenged with cardamom essential oil (CARD; white circles) via the drinking water starting on day 2 post-infection, whereas mock control mice received vehicle (black circles). On day 6 post-infection, the (**A**) small intestinal and (**B**) colonic lengths were measured with a ruler. Naive mice (open diamonds) served as untreated and uninfected controls. The box plots indicating the 75th and the 25th percentiles of the median (black bar within box), the total range, the levels of significance (*p* values) as calculated with the Kruskal–Wallis test and Dunn’s post-correction, and the number of analyzed mice (in parentheses) are shown. Data were pooled from four independent experiments.

Since gut inflammation might lead to shrinkage of the affected intestinal tissues [36], we measured the small and large intestinal lengths upon sacrifice (Figure 3). Mice from both treatment cohorts exhibited shorter colonic lengths as compared to naive control animals (*p* < 0.001), whereas the colons from the cardamom EO cohort were longer than those from the mock counterparts on day 6 p.i. (*p* < 0.001; Figure 3B). Of note, the small intestines did not differ in length between respective groups (n.s.; Figure 3A). Thus, cardamom EO treatment resulted in less distinct macroscopic inflammatory sequelae in *C. jejuni-*infected IL-10^−/−^ mice.

### 3.4. Histopathological and Apoptotic Responses in the Large Intestines Following Oral Cardamom EO Application to C. jejuni-Infected Microbiota-Depleted IL-10^−/−^ Mice

**Figure 4:**
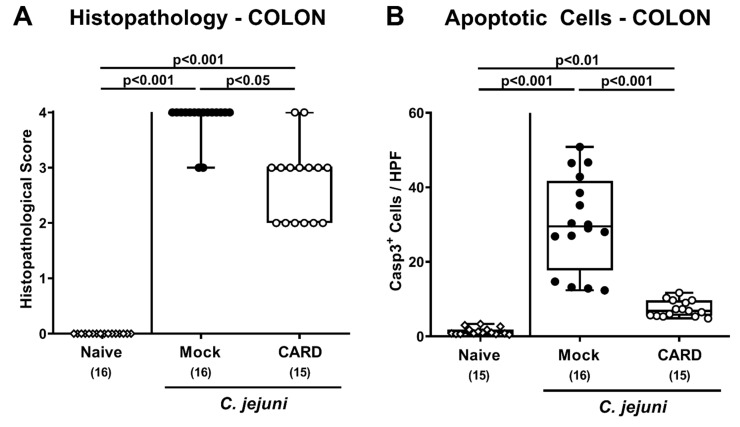
Histopathological and apoptotic responses in the large intestines following oral cardamom essential oil application to *C. jejuni*-infected microbiota-depleted IL-10^−/−^ mice. Microbiota-depleted IL-10^−/−^ mice were infected with *C. jejuni* strain 81–176 on days 0 and 1 by gavage and perorally challenged with cardamom essential oil (CARD; white circles) via the drinking water starting on day 2 post-infection, whereas mock control mice received vehicle (black circles). On day 6 post-infection, the (**A**) histopathological changes were quantitatively assessed in hematoxylin and eosin stained colonic paraffin sections, using a respective scoring system. In addition, (**B**) apoptotic colonic epithelial cells positive for cleaved caspase3 (Casp3^+^) were enumerated in immunohistochemically stained large intestinal paraffin sections (indicated are the median numbers of positively stained cells from six high power fields (HPF, 400× magnification) per animal). Naive mice (open diamonds) served as untreated and uninfected controls. The box plots indicating the 75th and the 25th percentiles of the median (black bar within box), the total range, the levels of significance (*p* values) as calculated with the Kruskal–Wallis test and Dunn’s post-correction, and the number of analyzed mice (in parentheses) are shown. Data were pooled from four independent experiments.

We next addressed whether cardamom EO treatment could also alleviate microscopic inflammatory changes upon *C. jejuni* infection and therefore surveyed histopathological changes in colonic paraffin sections. Whereas on day 6 p.i., mock treated mice displayed severe histopathological sequelae in their large intestines (*p* < 0.001), cardamom EO treated mice exhibited lower histopathological scores (*p* < 0.05; Figure 4A), indicative for moderate colonic intestinal inflammation. In addition, we quantified apoptotic epithelial cells in large intestinal paraffin sections and found increased numbers of colonic epithelial cells that were positive for cleaved caspase3 on day 6 p.i. of mock and cardamom EO treated mice (*p* < 0.01–0.001) but with much lower counts in the latter versus the former (*p* < 0.001; Figure 4B, Supplemental Figure S3A). Thus, cardamom EO treatment resulted in less distinct microscopic inflammatory sequelae in *C. jejuni-*infected IL-10^−/−^ mice.

### 3.5. Pro-Inflammatory Immune Responses in the Intestinal Tract Following Oral Cardamom EO Application to C. jejuni-Infected Microbiota-Depleted IL-10^−/−^ Mice

**Figure 5:**
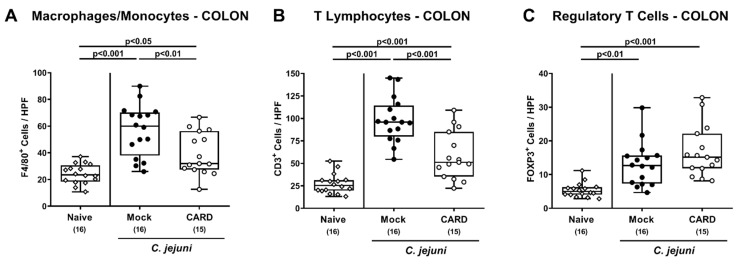
Immune cell responses in the large intestines following oral cardamom essential oil application to *C. jejuni*-infected microbiota-depleted IL-10^−/−^ mice. Microbiota-depleted IL-10^−/−^ mice were infected with *C. jejuni* strain 81–176 on days 0 and 1 by gavage and perorally challenged with cardamom essential oil (CARD; white circles) via the drinking water starting on day 2 post-infection, whereas mock control mice received vehicle (black circles). On day 6 post-infection, (**A**) macrophages and monocytes positive for cleaved F4/80, (**B**) T lymphocytes positive for CD3, and (**C**) regulatory T cells positive for FOXP3 were enumerated in the colonic mucosa and lamina propria within immunohistochemically stained large intestinal paraffin sections (indicated are the median numbers of positively stained cells from six high power fields (HPF, 400× magnification) per animal). Naive mice (open diamonds) served as untreated and uninfected controls. The box plots indicating the 75th and the 25th percentiles of the median (black bar within box), the total range, the levels of significance (*p* values) as calculated with the ANOVA test, and Tukey post-correction and the number of analyzed mice (in parentheses) are shown. Data were pooled from four independent experiments.

Next, we analyzed potential immune-modulatory effects of cardamom EO treatment during *C. jejuni* infection and therefore quantified distinct immune cell subsets following immunohistochemical staining of colonic paraffin sections. *C. jejuni* infection was associated with increased numbers of F4/80^+^ macrophages and monocytes, of CD3^+^ T lymphocytes and of FOXP3^+^ regulatory T cells in the colonic mucosa and lamina propria (*p* < 0.05–0.001; Figure 5, Supplemental Figure S3B–D), whereas caradamon EO treated mice displayed lower colonic numbers of macrophages, monocytes, and T lymphocytes as compared to mock control animals on day 6 p.i. (*p* < 0.01 and *p* < 0.001, respectively; Figure 5A,B, Supplemental Figure S3B,C).

**Figure 6:**
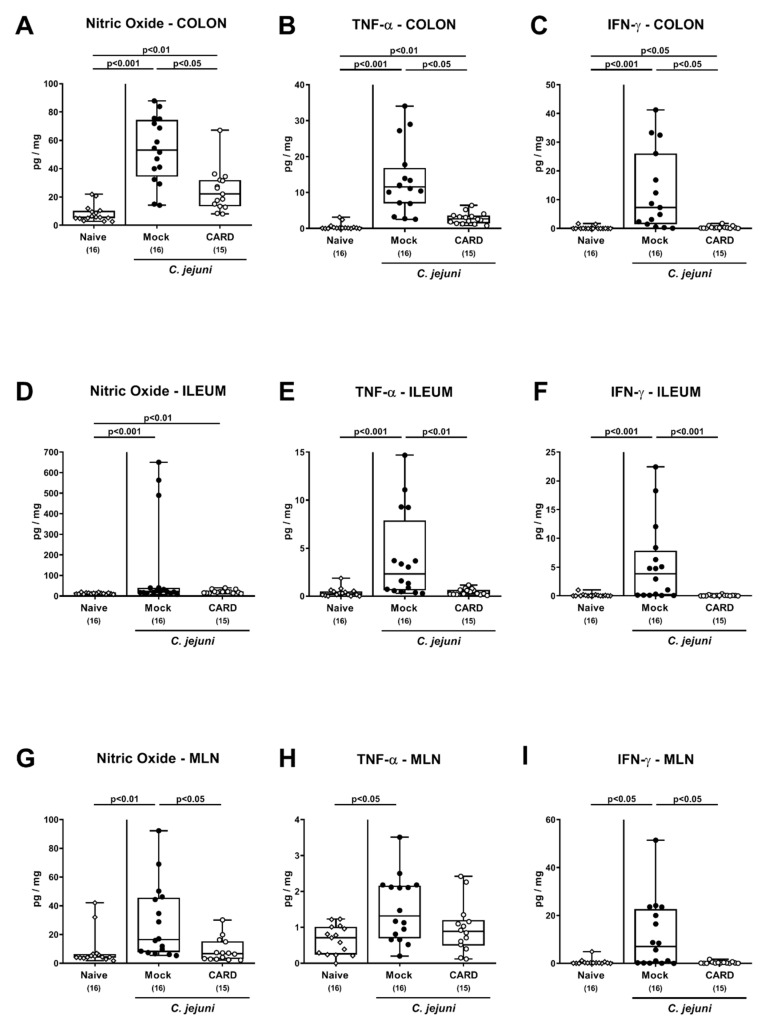
Intestinal pro-inflammatory mediator secretion following oral cardamom essential oil application to *C. jejuni*-infected microbiota-depleted IL-10^−/−^ mice. Microbiota-depleted IL-10^−/−^ mice were infected with *C. jejuni* strain 81–176 on days 0 and 1 by gavage and perorally challenged with cardamom essential oil (CARD; white circles) via the drinking water starting on day 2 post-infection, whereas mock control mice received vehicle (black circles). On day 6 post-infection, (**A**,**D**,**G**) nitric oxide, (**B**,**E**,**H**) TNF-α, and (**C**,**F**,**I**) IFN-γ concentrations were measured in ex vivo biopsies derived from the (**A**–**C**) colon, (**D**–**F**) ileum, and (**G**–**I**) mesenteric lymph nodes (MLN). Naive mice (open diamonds) served as untreated and uninfected controls. The box plots indicating the 75th and the 25th percentiles of the median (black bar within box), the total range, the levels of significance (*p* values) as calculated with the Kruskal–Wallis test and Dunn’s post-correction, and the number of analyzed mice (in parentheses) are shown. Data were pooled from four independent experiments.

We further assessed pro-inflammatory mediator secretion in distinct parts of the intestinal tract (Figure 6). Increased nitric oxide, TNF-α and IFN-γ concentrations were measured in colonic ex vivo biopsies derived from mock and cardamom EO treated mice on day 6 p.i. (*p* < 0.05–0.001), which were, however, lower in the latter as compared to the former (*p* < 0.05; Figure 6A–C). Furthermore, *C. jejuni* infection resulted in enhanced TNF-α and IFN-γ secretion in ileal samples derived from mock, but not cardamom EO treated mice (*p* < 0.001; Figure 6E,F), which also held true for nitric oxide, TNF-α, and IFN-γ concentrations assessed in MLN on day 6 p.i. (*p* < 0.05–0.001; Figure 6G–I). Thus, cardamom EO treatment resulted in diminished pro-inflammatory mediator secretion in the intestinal tract of *C. jejuni-*infected IL-10^−/−^ mice.

### 3.6. Extra-Intestinal IFN-γ Secretion Following Oral Cardamom EO Application to C. jejuni-Infected Microbiota-Depleted IL-10^−/−^ Mice

**Figure 7:**
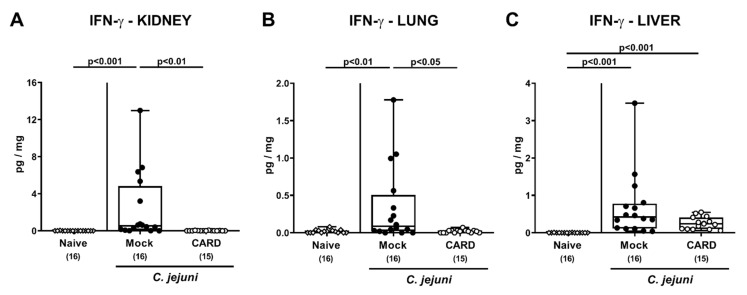
Extra-intestinal IFN-γ secretion following oral cardamom essential oil application to *C. jejuni*-infected microbiota-depleted IL-10^−/−^ mice. Microbiota-depleted IL-10^−/−^ mice were infected with *C. jejuni* strain 81–176 on days 0 and 1 by gavage and perorally challenged with cardamom essential oil (CARD; white circles) via the drinking water starting on day 2 post-infection, whereas mock control mice received vehicle (black circles). On day 6 post-infection, IFN-γ concentrations were measured in ex vivo biopsies derived from the (**A**) kidney, (**B**) lung, and (**C**) liver. Naive mice (open diamonds) served as untreated and uninfected controls. The box plots indicating the 75th and the 25th percentiles of the median (black bar within box), the total range, the levels of significance (*p* values) as calculated with the Kruskal–Wallis test and Dunn’s post-correction, and the number of analyzed mice (in parentheses) are shown. Data were pooled from four independent experiments.

We next addressed whether the observed immune-modulatory properties of cardamom EO treatment during *C. jejuni* infection was also effective beyond the intestinal tract. In fact, increased IFN-γ concentrations were measured in kidneys and lungs derived from mock as opposed to cardamom EO treated mice on day 6 p.i. (*p* < 0.001 and *p* < 0.01; Figure 7A,B). In liver ex vivo biopsies, however, *C. jejuni* infection was accompanied with enhanced IFN-γ secretion in mice of both cohorts (*p* < 0.001), but with a trend towards lower concentrations in cardamom EO versus mock treated mice on day 6 p.i. (n.s. given high standard deviations; Figure 7C).

**Figure 8:**
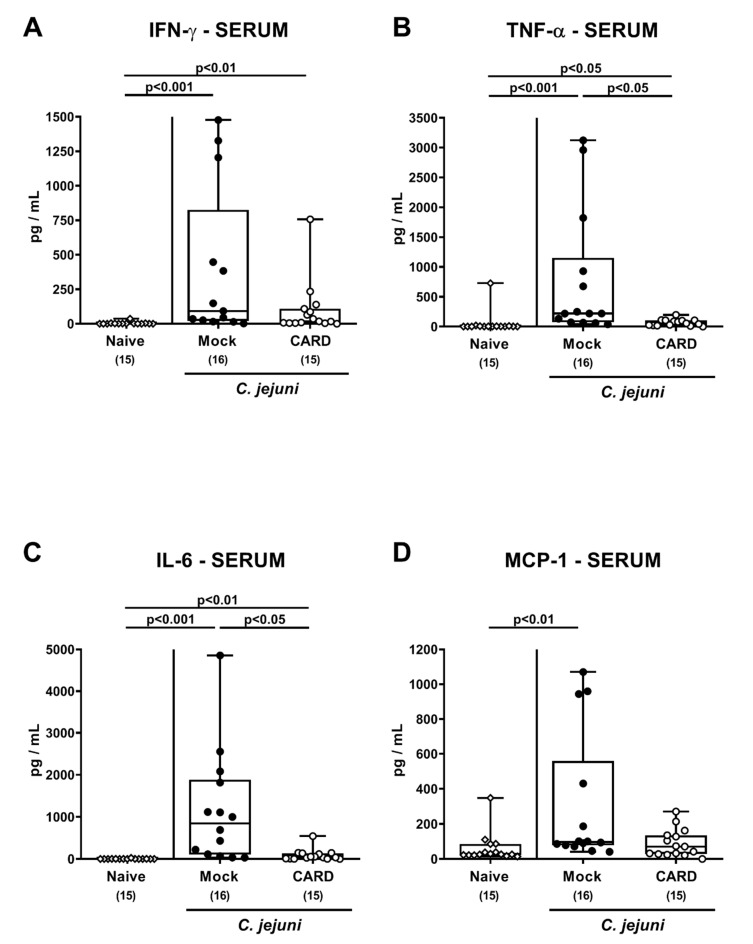
Systemic pro-inflammatory mediator secretion following oral cardamom essential oil application to *C. jejuni*-infected microbiota-depleted IL-10^−/−^ mice. Microbiota-depleted IL-10^−/−^ mice were infected with *C. jejuni* strain 81–176 on days 0 and 1 by gavage and perorally challenged with cardamom essential oil (CARD; white circles) via the drinking water starting on day 2 post-infection, whereas mock control mice received vehicle (black circles). On day 6 post-infection, (**A**) IFN-γ, (**B**) TNF-α, (**C**) IL-6, and (**D**) MCP-1 concentrations were measured in serum samples. Naive mice (open diamonds) served as untreated and uninfected controls. The box plots indicating the 75th and the 25th percentiles of the median (black bar within box), the total range, the levels of significance (*p* values) as calculated with the Kruskal–Wallis test and Dunn’s post-correction, and the number of analyzed mice (in parentheses) are shown. Data were pooled from four independent experiments.

When assessing systemic pro-inflammatory mediator secretion (Figure 8), *C. jejuni* induced increases in TNF-α and IL–6 serum concentrations were less pronounced upon cardamom EO as compared to mock treatment (*p* < 0.05; Figure 8B,C), whereas MCP-1 serum concentrations were elevated in the latter only (*p* < 0.01; Figure 8D). Furthermore, *C. jejuni* infection resulted in enhanced systemic IFN-γ secretion irrespective of the treatment regimen (0.01–0.001; Figure 8A). However, at least a trend towards lower IFN-γ serum concentrations could be assessed following cardamom EO as compared to mock treatment on day 6 p.i. (n.s. given high standard deviations; Figure 8A). Thus, cardamom EO treatment resulted in diminished pro-inflammatory mediator secretion in extra-intestinal and even systemic compartments of *C. jejuni-*infected IL-10^−/−^ mice.

### 3.7. Bacterial Translocation Following Oral Cardamom EO Application to C. jejuni-Infected Microbiota-Depleted IL-10^−/−^ Mice

We finally assessed whether viable *C. jejuni* might have translocated from the inflamed intestines to extra-intestinal tissue sites. Whereas bacterial translocation rates to the lungs and liver were comparable in mice from either cohort, *C. jejuni* could less frequently be isolated from the MLN (53.3% versus 75.0%), the kidneys (6.7% versus 12.5%), and the spleen (0.0% versus 18.8%) of cardamom EO as compared to mock treated mice on day 6 p.i. (Supplemental Figure S4). Of note, all blood samples remained *C. jejuni* culture-negative (Supplemental Figure S4F).

## 4. Discussion

Our present preclinical intervention study for the first time provides evidence that cardamom EO treatment of *C. jejuni*-infected microbiota-depleted IL-10^−/−^ mice resulted in lower intestinal pathogen burdens, improved clinical outcome, less distinct macroscopic and microscopic inflammatory sequelae, and in diminished pro-inflammatory mediator secretion not only in the intestinal tract, but also in extra-intestinal and, remarkably, even in systemic compartments during acute campylobacteriosis.

The pronounced disease-alleviating effects in *C. jejuni-*infected IL-10^−/−^ mice upon cardamom EO treatment could be observed as early as 24 hours after initiation of the treatment (i.e., day 3 p.i.). One might argue, however, that these beneficial effects of cardamom EO might have been due to its pathogen-lowering effects alone, given that on days 3 and 4 p.i., approximately two and one order of magnitude lower median fecal pathogen loads could be assessed in the verum versus the mock cohorts, respectively. At the end of the experiments, however, the median colonic *C. jejuni* counts were less than one order of magnitude lower in cardamom EO versus mock treated mice, which appears relatively subtle considering very high absolute pathogen numbers between 10^8^ and 10^9^ CFU per gram fecal sample. Nevertheless, in vitro analyses revealed antimicrobial effects of cardamom EO directed against *Campylobacter* including *C. jejuni* [35,43,44] by affecting bacterial membrane permeability, leading to cell lysis [44].

While mock control mice were suffering from acute enterocolitis, as indicated by severe wasting and bloody diarrhea, and were prone to succumb to infection, the clinical outcome in cardamom EO treated mice was much better and characterized by rather mild to moderate clinical signs. For instance, all mock controls were compromized by wasting symptoms, but this was true for only 13.3% of the animals from the cardamom EO cohort. Furthermore, 93.8% of the mice from the mock, but only 26.7% from the verum group suffered from diarrhea on day 6 p.i. In support, anti-diarrheal effects of cardamom extract have been shown in magnesium sulfate challenged rats previously [45].

Besides the improvement of the clinical outcome, cardamom EO treatment led to less distinct macroscopic inflammatory sequelae in *C. jejuni-*infected IL-10^−/−^ mice. This is indicated by less distinct shrinkage of the infected colon that was accompanied by less pronounced microscopic signs of acute enterocolitis such as lower histopathological scores and diminished apoptotic colonic epithelial cell responses in cardamom EO as compared to mock treated mice. In support, 1,8-cineole, a major constituent of cardamom EO, could effectively alleviate acute trinitrobenzene sulfonic acid (TNBS) induced acute colitis in rats [46], whereas the anti-apoptotic properties of cardamom extract was demonstrated in doxorubicin-induced cardiotoxicity in rats [47].

In our present study, cardamom EO treatment resulted in diminished pro-inflammatory immune responses upon *C. jejuni* infection of IL-10^−/−^ mice as indicated by less abundance of innate and adaptive immune cells such as macrophages and monocytes as well as T lymphocytes, respectively, in the colonic mucosa and lamina propria that was accompanied by less secretion of nitric oxide, TNF-α, and IFN-γ in the intestinal tract. Our data are supported by previous in vitro studies demonstrating potent anti-inflammatory effects of cardamom. For instance, cardamom treatment of murine splenocytes suppressed secretion of T helper cell-1 (Th1) cytokines including IFN-γ [31], which also held true for nitric oxide and TNF-α production by mouse peritoneal macrophages [26,31,48].

Remarkably, the potent inflammation-dampening effects of cardamom EO could also be observed beyond the intestinal tract, given accelerated *C. jejuni*-induced IFN-γ secretion in the kidneys and lungs of mock, but not cardamom EO treated mice. In support, cardamom was shown to exert renal-protective effects in gentamicin-induced nephrotoxicity in rats [49], whereas this compound has been used for the treatment of human kidney and urinary tract diseases in traditional medicine for a long time [28,50]. Furthermore, 1,8-cineole, a major cardamom EO constituent, suppressed inflammatory responses in alveolar macrophages in vitro [51], and oral cardamom application even protected Swiss mice from Pan masala induced lung damage [52].

In our study, a trend towards lower hepatic IFN-γ concentrations could be observed in cardamom EO versus mock treated mice on day 6 p.i. even though not reaching statistical significance. Nevertheless, cardamom improved hepatic function in alcoholic liver damage [53], provided hepatoprotective effects on experimental liver injuries of different etiologies [54,55,56], and of note, a controlled clinical trial provided evidence for beneficial effects of cardamom application to overweight and obese subjects with fatty liver disease [57].

Of note, viable *C. jejuni* translocated less frequently from the inflamed intestines to the MLN and to extra-intestinal organs such as the kidneys and the spleen derived from cardamon EO as compared to mock treated mice on day 6 p.i., pointing towards a less compromized epithelial barrier function in the former versus the latter.

Strikingly, the potent anti-inflammatory effects of oral cardamom EO application to mice suffering from acute campylobacteriosis were also effective systemically as indicated by lower TNF-α, IL-6, and MCP-1 concentrations measured in serum samples taken from verum versus mock mice on day 6 p.i. We would like to further emphasize that the four-day cardamom EO treatment period was relatively short. It is tempting to speculate that the beneficial effects might have been even more pronounced upon initiation of the oral cardamom EO application before enterocolitis induction. This prophylactic approach will be addressed in future studies.

## 5. Conclusions

In conclusion, our preclinical intervention study provides evidence for the first time that cardamom EO comprises a promising compound for the combat of acute campylobacteriosis and presumably prevention of post-infectious morbidities. Given the relative safe profile and long use of this compound in traditional medicine, cardamom EO paves the way to clinical studies with humans suffering from campylobacteriosis in order to tackle this acute inflammatory syndrome in an antibiotics-independent, immune-modulatory fashion.

## Supplementary Materials

The following are available online at https://www.mdpi.com/2076-2607/9/1/169/s1: Figure S1: Fecal *C. jejuni* loads over time following oral cardomon essential oil application to *C. jejuni*-infected microbiota-depleted IL-10^−/−^ mice. Figure S2: Clinical conditions over time following oral cardomon essential oil application to *C. jejuni*-infected microbiota-depleted IL-10^−/−^ mice. Figure S3: Representative photomicrographs of immunohistochemically stained colonic paraffin sections. Figure S4: Bacterial translocation following oral cardomon essential oil application to *C. jejuni*-infected microbiota-depleted IL-10^−/−^ mice.
