# Supplementary material for: Anti-Pathogenic and Immune-Modulatory Effects of Peroral Treatment with Cardamom Essential Oil in Acute Murine Campylobacteriosis
Source: Microorganisms. 2021 Jan 14;9(1):169. doi: 10.3390/microorganisms9010169 (PMC7828794; doi:10.3390/microorganisms9010169)
Supplement: Supplementary file 1 [file microorganisms-09-00169-s001.zip › Supp Fig 3_PICS_CARDAMON_COLON_09.01.21.pdf]

A Apoptotic Cells - COLON

Naive **Mock** **Cardamom EO**

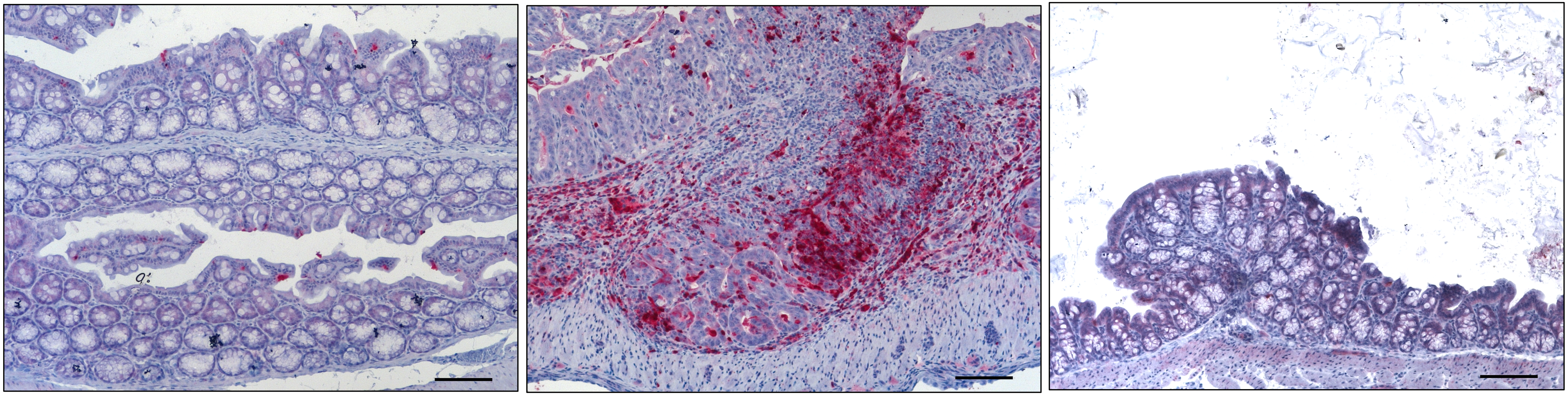

(100 x magnification, scale bar 100 μm)

B Macrophages / Monocytes - COLON

Naive **Mock** **Cardamom EO**

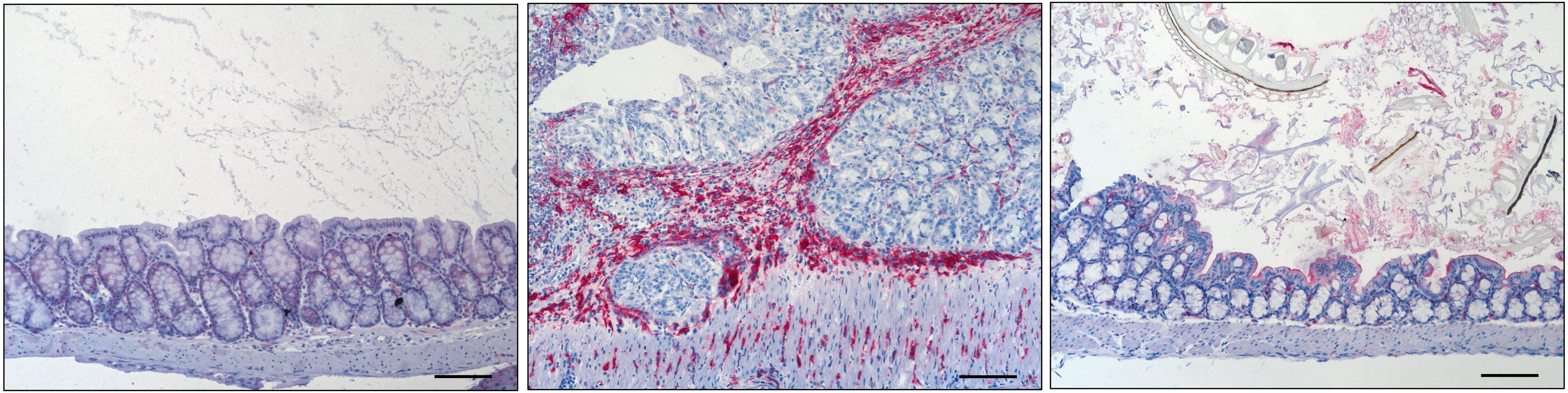

(100 x magnification, scale bar 100 µm)

C T Lymphocytes - COLON

Naive Mock Cardamom EO

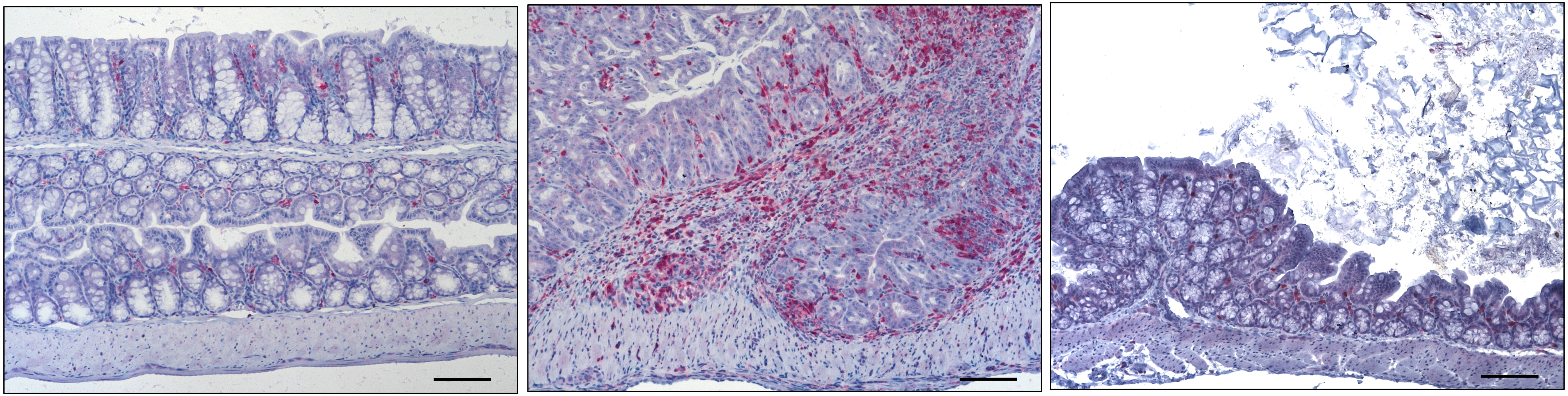

(100 x magnification, scale bar 100 µm)

D Regulatory T Cells - COLON

Naive | Mock | Cardamom EO

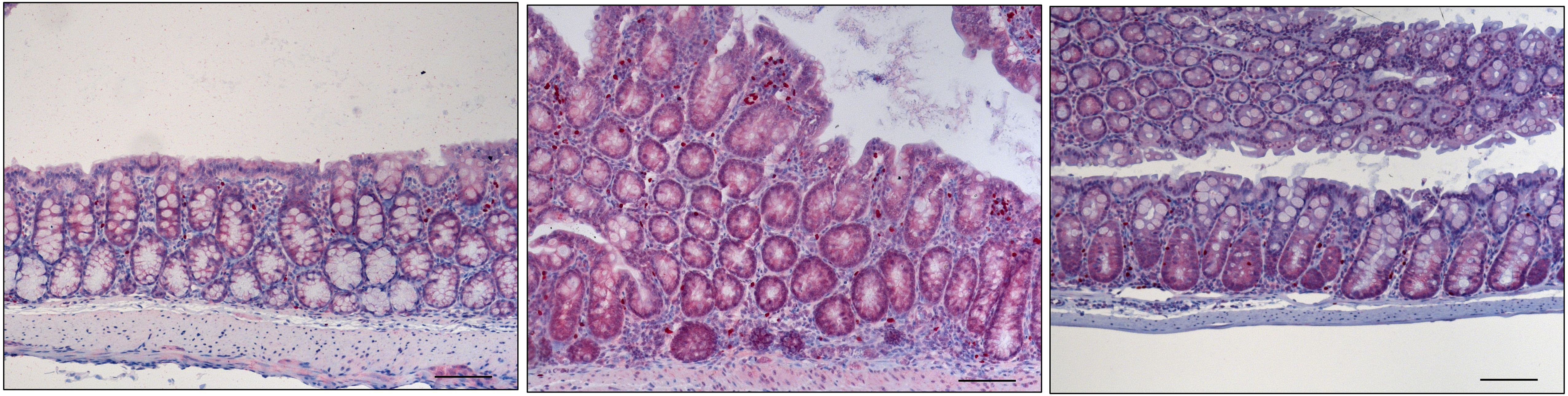

(100 x magnification, scale bar 100 µm)
