# Supplementary figures and images for: Anti-Pathogenic and Immune-Modulatory Effects of Peroral Treatment with Cardamom Essential Oil in Acute Murine Campylobacteriosis
Source: Microorganisms. 2021 Jan 14;9(1):169. doi: 10.3390/microorganisms9010169 (PMC7828794; doi:10.3390/microorganisms9010169)

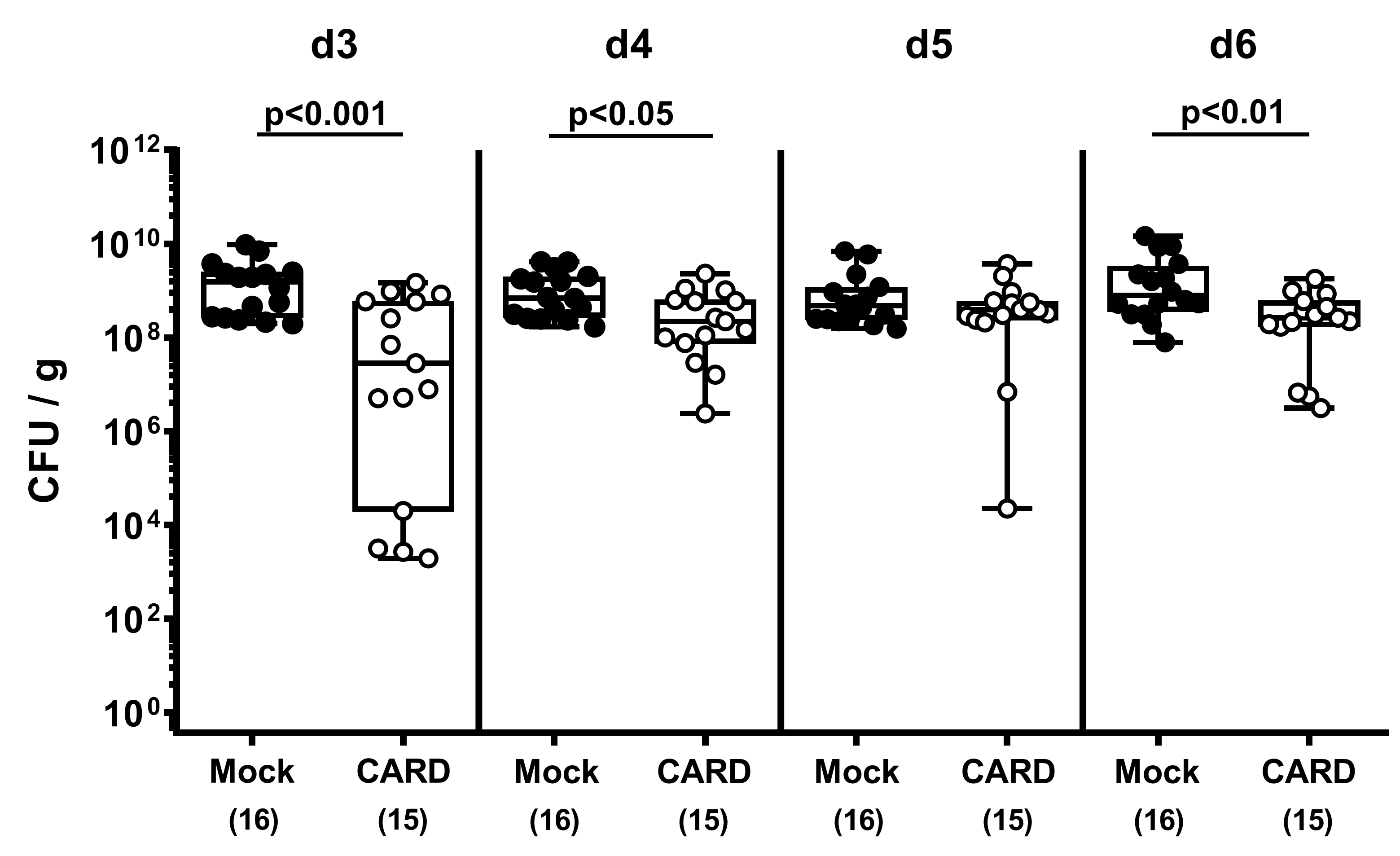

Supplement: Supplementary file 1 [file microorganisms-09-00169-s001.zip › Supp Fig 1_ Placebo vs CARD.tif]

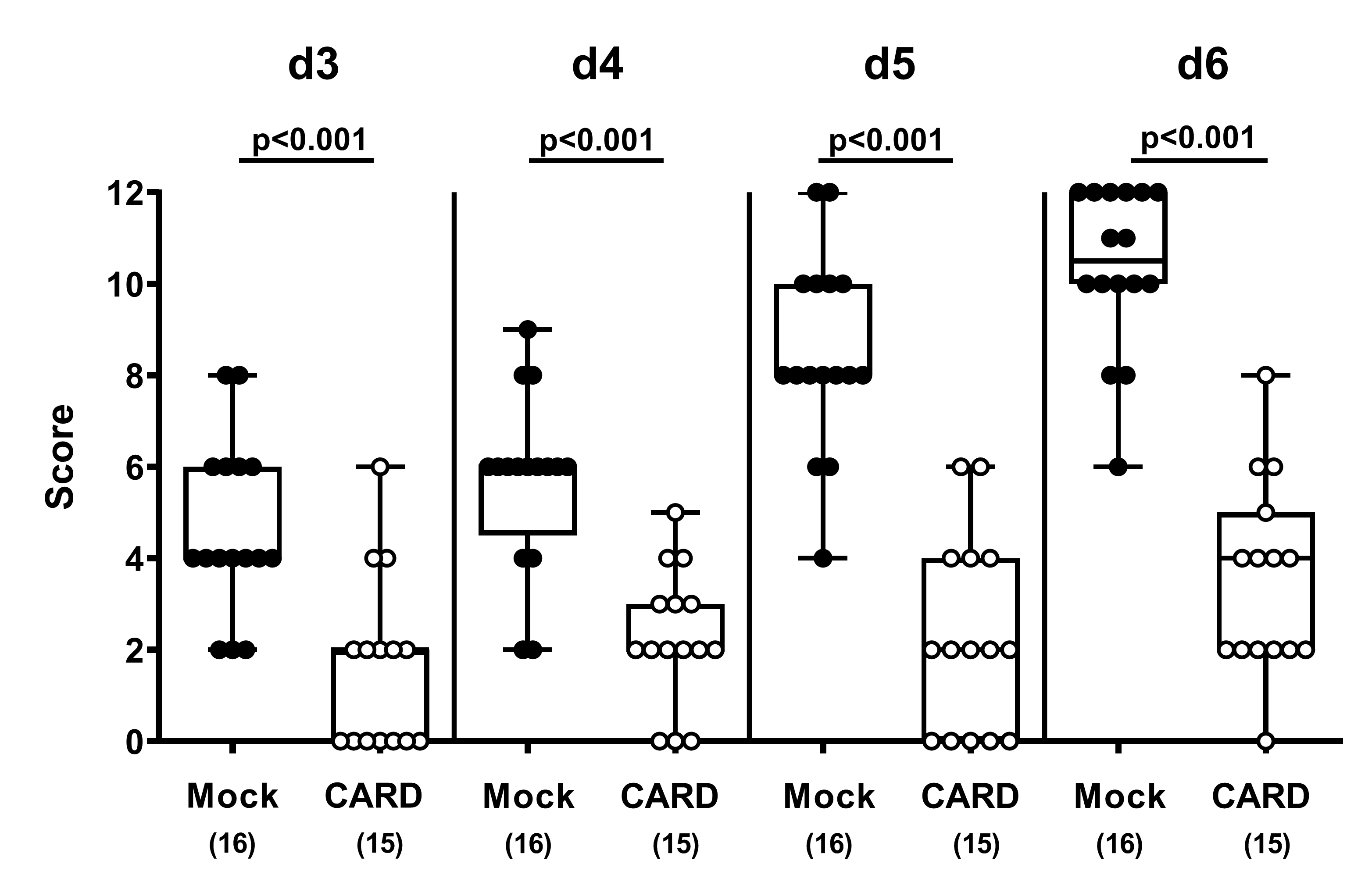

Supplement: Supplementary file 1 [file microorganisms-09-00169-s001.zip › Supp Fig 2_Total Clinical Score d3-d6.tif]

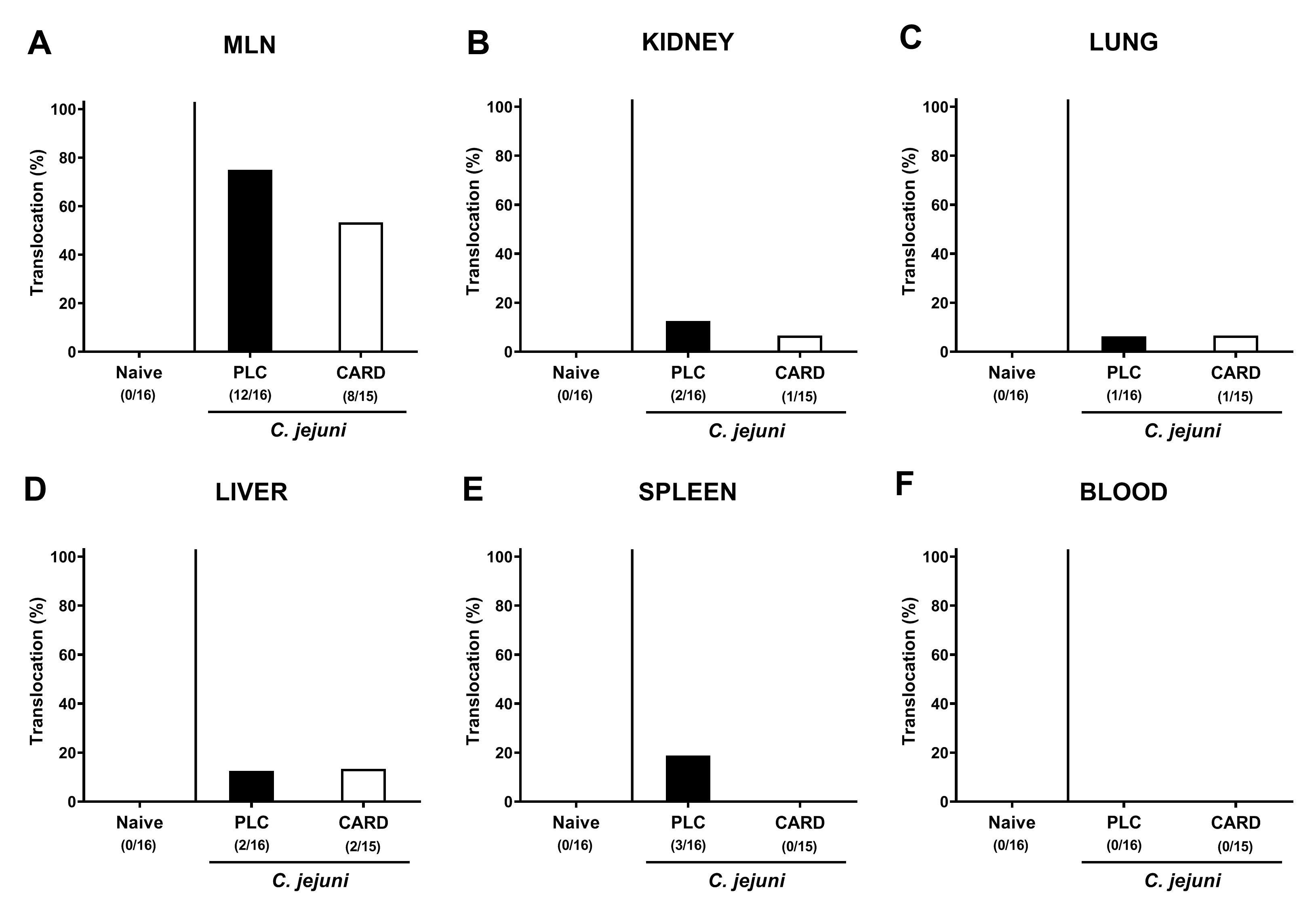

Supplement: Supplementary file 1 [file microorganisms-09-00169-s001.zip › Supp Fig 4_Translokation_Cardamom.tif]
